# Hyperglycemia as a risk factor for the development of retinopathy of prematurity

**DOI:** 10.1186/1471-2431-13-78

**Published:** 2013-05-16

**Authors:** Shakir Mohamed, Jeffrey C Murray, John M Dagle, Tarah Colaizy

**Affiliations:** 1 Department of Pediatrics, Division of Newborn Medicine, Washington University in St. Louis, 660 S. Euclid Ave, Campus Box 8116, St. Louis, MO 63110, USA; 2 Department of Pediatrics, University of Iowa, Iowa City, IA 52242, USA

**Keywords:** Prematurity, Retinopathy of prematurity, Hyperglycemia

## Abstract

**Background:**

Hyperglycemia has recently been described as a risk factor for the development of retinopathy of prematurity (ROP), a proliferative vascular disease of the retina that primarily affects premature infants. This study was to evaluate the relationship of hyperglycemia and the development of ROP in premature infants less than 32 weeks gestation.

**Methods:**

This was a retrospective cohort study of all infants less than 32 weeks gestation from 2003–2007 who survived to discharge in our NICU. Demographic data including birthweight, gestational age, Apgar scores, method of delivery, antenatal steroid use, neonatal steroid use, and size for gestational age was collected for each infant. Episodes of sepsis, grade of intraventricular hemorrhage, presence of a patent ductus arteriosus, number of days on the ventilator, and stage of necrotizing enterocolitis were assessed as well as days of hyperglycemia, defined as number of days with whole blood glucose > 150 mg/dl. In addition, the highest stage of ROP was recorded for each infant. A Student’s two tailed t-test or Fisher’s exact test was performed to identify significant clinical risk factors associated with the development of ROP. From this univariate analysis, a multiple logistic regression was performed to determine the effect of hyperglycemia on the development of ROP, adjusting for significant clinical risk factors. Statistical analysis was performed using SAS v.9.2.

**Results:**

Univariate analysis demonstrated that infants with ROP were of lower birthweight and gestational age, and were affected by a patent ductus arteriosus, neonatal sepsis, intraventricular hemorrhage, have significant lung disease and received postnatal glucocorticoid therapy. Infants with ROP experienced more days with hyperglycemia (7 vs. 2, p = < 0.0001). Using multiple logistic regression analysis to compare no ROP vs. all stages of ROP, gestational age (OR 0.745, 95% CI [0.634, 0.877], p = 0.0004), mean days of hyperglycemia (OR 1.073, 95% CI [1.004, 1.146], p = 0.04), and mean days receiving mechanical ventilation (OR 1.012, 95% CI [1.000, 1.025], p = 0.05) remained significantly associated with ROP after adjusting for other risk factors.

**Conclusion:**

Our data suggests that hyperglycemia is associated with the development of ROP in premature infants.

## Background

Retinopathy of prematurity (ROP) is a proliferative vascular disease of the retina that primarily affects premature infants and is one of the leading causes of visual loss in children [1]. Premature infants that are born at low birth weight or at a lower gestational age are particularly vulnerable [2].

In addition to genetic risk factors that are associated with the development of ROP [3-5], there are well established environmental risk factors for ROP, including early oxygen exposure and extreme prematurity [6]. Hyperglycemia is a newly reported environmental risk factor for ROP. Hyperglycemia, commonly defined as whole blood glucose level greater than 125 mg/dl or plasma glucose level greater than 150 mg/dl, occurs frequently in premature infants. In a study by Dweck *et al.*, it was found that 86% of infants with a birthweight less than 1100 grams were hyperglycemic, with the highest risk of being hyperglycemic occurring within the first 24 hours of birth [7]. Data from a cohort analyses of the NIRTURE study also demonstrated that 80% of very low birth weight infants had glucose levels > 8 mmol/L (>144 mg/dL) and 32% had glucose levels > 10 mmol/L (>180 mg/dL) >10% of the time in the first week of life [8].

Recent studies have shown an association between neonatal hyperglycemia and (1) increased risk of early death and morbidity in extremely low birth weight infants [9,10], (2) increased morbidity and mortality in premature infants with necrotizing enterocolitis [11], and (3) has also been shown to be a risk factor for death and white matter reduction in preterm infants [12]. In addition, previous studies have demonstrated an association between hyperglycemia and the development of ROP [13-15]. The purpose of this study was to evaluate the relationship between the duration of hyperglycemia and the development of ROP in a larger cohort of premature infants born at less than 32 weeks gestation.

## Methods

We performed a retrospective cohort study examining the relationship between hyperglycemia and development of ROP, adjusting for multiple risk factors. A retrospective chart review was performed of all infants less than 32 weeks gestational age admitted to the Neonatal Intensive Care Unit at the University of Iowa Children’s Hospital from 2003–2007 who had ophthalmologic screening for ROP. Initial ophthalmologic screening was performed on all patients with gestational age less than 32 weeks or birthweight less than 1500 gram and follow up was at the discretion of the ophthalmologist. ROP severity was staged by the International Classification of ROP [16] and the highest stage of ROP was recorded.

Subject demographic information (birth weight, gestational age, Apgar scores, gender, method of delivery, antenatal steroid use, neonatal steroid use, size for gestational age) was extracted from the patient’s medical records. Episodes of sepsis, presence of intraventricular hemorrhage (IVH), presence of a patent ductus arteriosus (PDA), necrotizing enterocolitis (NEC, ≥ Bell’s Stage II) [17], and number of days of ventilator support were recorded as well. Sepsis was defined as any positive culture (blood, urine, or cerebrospinal fluid) that occurred at any point during the hospital stay. IVH was graded by the attending pediatric radiologist and the highest grade was recorded. The presence of a PDA was determined by standard echocardiography performed by a pediatric cardiologist according to our NICU clinical guidelines. All infants < 28 weeks gestation were evaluated for PDA between days of life 5 through 7 regardless of symptoms, and infants ≥ 28 weeks gestation were evaluated between days of life 5 through 7 if the infant had a murmur consistent with a PDA.

In this study, hyperglycemia was defined as the number of days with at least one whole blood glucose level greater than 150 mg/dl. Glucose levels were determined from whole blood using the glucose oxidation technique (ABL800 Flex, Radiometer America Inc., USA). Days of hyperglycemia was chosen for analysis rather than highest recorded glucose level to minimize confounding due to differences in the number of laboratory measurements obtained in smaller, sicker infants as compared to larger, healthier preterm infants.

Initial univariate analysis was performed to evaluate individual clinical risk factors associated with the development of ROP utilizing either a Student’s two tailed t-test for comparison of means or Fisher’s exact test for comparison of categorical values. From this initial univariate analysis, a multiple logistic regression analysis was performed to determine the effect of hyperglycemia on the development of ROP, adjusted for significant clinical risk factors. We first modeled the relationship between of any degree of ROP vs. no ROP, adjusting for clinical factors found to be associated with ROP in univariate analyses. This was the primary outcome of our study. In addition, further analyses were performed to evaluate the relationship of hyperglycemia to different degrees of ROP. All statistical analysis was performed using SAS v.9.2 (SAS Institute Inc., Cary, NC, USA). Approval of the University of Iowa Institutional Review Board was obtained to access the clinical information required for this study.

## Results

We identified 582 infants less than 32 weeks gestation that underwent ophthalmologic screening for ROP. Overall, 412 infants had no ROP and 170 had ROP (57 with Stage I, 65 with Stage II, and 48 with Stage III). There were no cases of Stage IV or V ROP in this study cohort. No infants in this trial received insulin therapy for hyperglycemia.

### Univariate analyses

Table 1 outlines the results of the univariate analysis. Not surprisingly, infants diagnosed with ROP were of lower birthweight and gestational age compared to those without ROP, and were more likely to have been affected by a PDA, neonatal sepsis, and intraventricular hemorrhage. Infants with ROP were also more likely to have significant lung disease and to have received postnatal glucocorticoid therapy than those without ROP. There were no differences between ROP and non-ROP subjects with respect to rate of c-section delivery, gender, or NEC. Infants with ROP experienced a greater mean number of days with hyperglycemia (7 vs. 2, p = < 0.0001).

**Table 1:** Clinical characteristics of study population

	**No ROP**	**Any ROP**	**p-value**
	**(n = 412)**	**(n = 170)**	
Birthweight gm (mean)	1080 ± 272	831 ± 266	< 0.0001
Gestational age, weeks (mean)	28.1 ± 1.8	25.8 ± 1.9	< 0.0001
One minute APGAR (mean)	5.5 ± 2.3	4.4 ±2.2	< 0.0001
Five minute APGAR (mean)	7.5 ± 1.5	6.5 ± 1.9	< 0.0001
Male gender (%)	50.5	53.5	0.52
C-section (%)	72.8	67.1	0.19
Antenatal steroids (%)	90.7	87.3	0.15
Small for gestational age (%)	15.6	13.5	0.53
Patent ductus arteriosus (%)	35.2	66.5	< 0.0001
Intraventricular hemorrhage (%)	18.2	31.8	< 0.0006
Necrotizing enterocolitis (%)	2.9	4.1	0.62
Neonatal sepsis (%)	20.6	42.4	< 0.0001
Neonatal steroid therapy (%)	14.6	50	< 0.0001
Hyperglycemia days (mean)	2.3 ± 3.2	7.1 ± 6.6	< 0.0001
Ventilator days (mean)	17.0 ± 20.6	49.6 ± 47.7	< 0.0001

### Multiple logistic regression

From this univariate analysis, a multiple logistic regression analysis was performed to analyze the association of hyperglycemia with ROP while adjusting for other significant risk factors as shown in Tables 2, 3. We first modeled ROP as any ROP vs. no ROP, including the whole study population. In Table 2, when comparing no ROP vs. all stages of ROP, gestational age (OR 0.745, 95% CI [0.634, 0.877], p = 0.0004), days of hyperglycemia (OR 1.073, 95% CI [1.004, 1.146), p = 0.04], and days receiving mechanical ventilation (OR 1.012, 95% CI [1.000, 1.025], p = 0.05) remained significantly associated with ROP after adjusting for other risk factors. Specific tests of multicollinearity were not performed. The risk of being diagnosed with ROP increased 7% for each additional day of hyperglycemia.

**Table 2:** Risk factors for the development of ROP, n = 582*

**Clinical variable**	**Beta**	**Odds**	**95% CI**	**p-value**
	**coeff**	**ratio**		
**Gestational age , wks**	**−0.29**	**0.745**	**(0.634, 0.877)**	**0.0004**
**Hyperglycemia days**	**0.07**	**1.073**	**(1.004, 1.146)**	**0.04**
**Ventilator days**	**0.01**	**1.012**	**(1.000, 1.025)**	**0.05**
Sepsis	0.18	1.195	(0.784, 1.823)	0.41
IVH	0.47	1.608	(0.975, 2.652)	0.06
PDA	0.12	1.130	(0.892, 1.432	0.31
Neonatal steroids	0.43	1.537	(0.904, 2.613)	0.11

For all dichotomous predictor variables 1 = presence of risk factor, 0 = absence of risk factor.

* no ROP = 0, any ROP =1, probability modeled is ROP = 1.

**Table 3:** Risk factors for development of stage 3 ROP among all infants, n = 582*

**Clinical variable**	**Beta coeff**	**Odds ratio**	**95% CI**	**p-value**
**Gestational age**	**−0.37**	**0.690**	**(0.533, 0.892)**	**0.04**
Hyperglycemia days	0.0003	1.000	(0.938, 1.066)	0.99
**Ventilator days**	**0.0134**	**1.013**	**(1.001, 1.027)**	**0.04**
Sepsis	0.562	1.756	(0.915, 3.4)	0.09
**IVH**	**0.716**	**2.045**	**(1.017, 4.113)**	**0.04**
PDA	0.035	1.036	(0.702, 1.528)	0.86
Neonatal steroids	−0.072	0.931	(0.422, 2.054)	0.86

For all dichotomous predictor variables 1 = presence of risk factor, 0 = absence of risk factor.

* no ROP, stage 1 or 2 ROP = 0, stage 3 ROP =1, probability modeled is stage 3 ROP = 1.

We then undertook further analyses to explore the relationship of hyperglycemia to different degrees of ROP. We modeled risk factors associated with development of severe (Stage III) ROP among all infants (Table 3). Gestational age (OR 0.69, 95% CI [0.53, 0.90], p = 0.005), ventilator days (OR 1.01, 95% CI [1.001, 1.027] p = 0.04), and IVH (OR 2.0, 95% CI [1.01, 4.1] p = 0.044) were significantly associated with ROP. We then modeled development of severe ROP only among those infants who developed any ROP (170 infants), and no significant risk factors were identified. In neither model was hyperglycemia a risk factor for the development of severe ROP.

## Discussion

This study demonstrates an association between duration of hyperglycemia (defined as number of days with whole blood glucose > 150 mg/dL) and the development of ROP in premature infants less than 32 weeks gestational age.

There is emerging evidence that neonatal hyperglycemia is a significant risk factor in neonatal morbidity and mortality. In a study by Kao *et al.* evaluating hyperglycemia and morbidity and mortality in extremely low birth weight (ELBW) infants, it was seen that there was a relationship between severe hyperglycemia (serum glucose > 180 mg/dL) and an increased risk of death or sepsis [10]. In another study by Hays *et al.*, also evaluating hyperglycemia as a risk factor for early death and morbidity in ELBW infants, it was shown that neonatal hyperglycemia was significantly associated with an increased risk of death or the development of Grade III/IV IVH [9].

Hyperglycemia has also been previously shown to be associated with the development of ROP. In a study by Garg *et al.* evaluating hyperglycemia and the development of Stage III or IV ROP, it was shown that for each 10 mg/dL increase of mean serum glucose, there was 2.7-fold increase in the risk of developing ROP [14]. Blanco *et al.* also found that hyperglycemia was associated with a 4.5-fold increase in the incidence of ROP in a Hispanic population of ELBW infants [18]. In a recent study by Kaempf *et al.*, hyperglycemia and insulin exposure was shown to be associated with higher stages of ROP [15]. In our study, we have shown that the duration of hyperglycemia is a significant risk factor in the development of ROP, with each day of hyperglycemia increasing the risk by 7% in a population of ELBW infants who did not receive insulin therapy. Studying infants who did not receive insulin therapy allowed us to investigate the role of hyperglycemia in the incidence of ROP independently of insulin exposure. Our study also shows that duration of hyperglycemia is associated with ROP, building on previous studies that chose highest glucose at one time point to define hyperglycemia.

Interestingly, however, we were unable to establish an association between duration of hyperglycemia and severe (Stage III) ROP. Utilizing two different multiple logistic regression models, duration of hyperglycemia was found to not be associated with the development of severe (Stage III) ROP. This is in contrast to Garg *et al’s* study, where hyperglycemia, as defined by highest measured blood glucose in the first month of life, was found to be associated with the development of Stage III or IV ROP, although they specifically chose to study the impact of glucose on Stages III and IV and included infants with Stage I in the control group of their case–control study. Similar to our results, they also demonstrated a significantly higher number of days with glucose measurements of > 150 mg/dl among patients with ROP (8.4 days vs. 5.3 days, p = 0.028), but did not include duration of hyperglycemia in multivariable analysis [14].

In adults, hyperglycemia has been shown to play a significant role in the development of proliferative diabetic retinopathy [19]. Like ROP, proliferative diabetic retinopathy is characterized by the development of new blood vessels in the retina that can extend into the vitreous of the eye. Additionally, similar to severe ROP, retinal detachment can occur due to the fibrous contractile tissue that is formed [20].

Hyperglycemia may influence ROP through its significant effect on retinal blood flow [21]. In diabetic rats, reduction of glucose levels resulted in an improvement in retinal blood flow when compared to diabetic rats who remained hyperglycemic [22]. Additionally, hyperglycemia has been shown to increase the formation of diacylglycerol, which in turn increases activation of protein kinase C. Protein kinase C has been shown to have an effect on many different growth factors, such as vascular endothelial growth factor (VEGF), which impacts angiogenesis and vascular permeability [23]. Hyperglycemia, in hypoxic culture conditions, has also been shown to increase VEGF production in retinal Müller cells [24]. Additionally, in an *in vitro* assay of VEGF production in cultured bovine retinal pigmented epithelial cells, it was shown that those cells that were exposed to a prolonged period of hyperglycemia had significantly elevated level of VEGF production when compared to controls [25]. While the factors that lead to the progression of ROP are not well understood, it is possible that the duration of hyperglycemia is a factor in the initiation of ROP, but other factors such as oxygen exposure and genetics have a more critical role in influencing the factors that are involved in the progression of the disease. As such, duration of hyperglycemia could potentially be more of a risk factor for the development of mild (Stage I) or moderate (Stage II) ROP rather than severe (Stage III) ROP.

Our study is limited due to its retrospective nature. Although we have shown an association between hyperglycemia and the development of ROP, we have not presented evidence that is suggestive of causation. The question remains whether hyperglycemia itself is a definitive risk factor, or is a marker of significant illness. We did adjust for other known risk factors which are markers for more severe illness, but the possibility of residual confounders is likely. However, using proliferative diabetic retinopathy as a model of a proliferative vascular retinal disease, it has been shown that hyperglycemia has a significant impact on the development and progression of the disease.

## Conclusion

In conclusion, we have shown an association between duration of hyperglycemia and the development of ROP in premature infants less than 32 weeks gestation, adjusting for multiple risk factors. As hyperglycemia is a partially modifiable risk factor, further prospective studies are needed to fully evaluate the impact of hyperglycemia and the development of ROP.

## Abbreviations

ROP: Retinopathy of prematurity; IVH: Intraventricular hemorrhage; PDA: Patent ductus arteriosus; NEC: Necrotizing enterocolitis.

## Competing interests

The authors declare that they have no competing interests.

## Authors’ contributions

SM was involved in chart review, data synthesis, statistical analysis and drafted the manuscript. JCM and JMD were involved in study design and editing the manuscript. TC was involved in study design, statistical analysis, editing the manuscript, and oversaw the study. All authors have read and approved this manuscript.

## Pre-publication history

The pre-publication history for this paper can be accessed here:

http://www.biomedcentral.com/1471-2431/13/78/prepub
